# Antimicrobial Peptides: A Potential Therapeutic Option for Surgical Site Infections

**Published:** 2017-11-16

**Authors:** Berthony Deslouches, Y Peter Di

**Affiliations:** 1 Department of Environmental and Occupational Health, University of Pittsburgh Graduate School of Public Health, Pittsburgh, PA, USA; 2 Department of Microbiology and Molecular Genetics, University of Pittsburgh School of Medicine, Pittsburgh, PA, USA

## Abstract

Surgical Site Infections (SSI) represents one of the most common hospital-associated infections worldwide, and many cases of SSI are due to multidrug-resistant bacteria with the propensity to attach to tissues and form biofilm on post-surgical sites. While systemic antibiotic treatment (prophylactically and therapeutically) is usually effective, SSI can be difficult to treat when associated with drug resistance. Antimicrobial Peptides (AMPs) are an untapped resource that could serve as an effective therapeutic option, as they display broad-spectrum antimicrobial activity regardless of antibiotic resistance. In the last decade, it has become increasingly clear that AMPs also display antibiofilm properties. We reviewed herein the potential of AMPs as promising therapeutics for SSI and the need for structural optimization to develop AMPs for clinical applications.

## Introduction

Natural and Synthetic Antimicrobial Peptides (AMPs) exhibit great potential as new therapeutic agents for surgical site infections. AMPs rapidly kill their bacterial targets by membrane-disruption, or less commonly by interference with vital intracellular functions (e.g., DNA, RNA, protein synthesis) [1-3]. AMPs also display anti-biofilm activities [4,5]. However, after more than three decades of AMP discoveries, they are yet to be clinically established as practical antibiotics against common Multi-Drug Resistant (MDR) bacteria. The alarming emergence of MDR pathogenic microorganisms [6-8], coupled with a decrease in the pharmaceutical industry research pipeline for novel antimicrobial agents, has created an urgent need to develop new strategies to address the pressing problems associated with current infectious diseases [9]. Thus, infections associated with MDR pathogens constitute an imminent health crisis (World Health Organization, 2014) [6,10]. These infections are of particular importance to surgical patients, as hospital-associated transmission of MDR bacteria in these patients can frequently lead to life-threatening complications [11-13]. Surgical Site Infections (SSI) refer to infections of the skin and subcutaneous or deep tissues that occur at a manipulated site within 30 days after an operation (WHO, 2016) [14,15]. The mean incidence of SSI is 7 to 15 cases per 100 hospitalized patients, with higher incidence in lower-income nations. However, even in higher-income countries, SSIs remain the most or second most common nosocomial infections [15]. The most frequent etiologic agents are *Staphylococcus aureus* (30%) and coagulase-negative staphylococci (12%), which are associated with the skin flora. Other causative agents include the Enterobacteriaceae *Escherichia coli, Enterobacter spp*, and *Klebsiella pneumoniae* as well as *Enterococcus faecalis and Pseudomonas aeruginosa*, which all belong to the group of MDR bacteria referred to as ESKAPE pathogens [14-17]. In addition to the propensity to develop drug resistance, when attached to tissues, these organisms tend to adopt a biofilm mode of growth [18], which usually exacerbates the progression of the wound to a chronic, non-healing state eventually resulting in the sequelae of sepsis [19]. Importantly, biofilm tends to be inherently resistant to current antibiotics [5,20]. We and other investigators have postulated that AMPs possess unique properties that warrant their development as potential countermeasures to combat MDR pathogens.

## Properties of Natural AMPs

Most AMPs are short cationic peptides (10-50 amino acids long) with an amphipathic (cationic and hydrophobic domains) structure that are ribosomally synthesized in most life forms, including humans [21-26]. Hence, ubiquitous in nature, AMPs represent the first line of defense against a variety of microbial pathogens (*e.g.*, bacteria, fungi, parasites, viruses) [5,27-33]. AMPs are structurally diverse (α-helix, β-sheets, loop structures; reviewed in detail elsewhere) [32-36], with the amphipathic structure as a consensus motif required for antimicrobial activity [37]. AMPs generally recognize their bacterial targets via electrostatic interactions with negatively charged bacterial membrane lipids [38-43]. For the α-helical AMPs in particular (e.g., the human helical AMP LL37) [4,22,44-46], these membrane interactions are required for induction of the secondary structure, which is random coil in aqueous solutions. Although bacterial killing commonly occurs via membrane perturbation mechanisms (e.g., LL37, magainin), other antimicrobial mechanisms of AMPs have been demonstrated including bacterial cell penetration (e.g., proline-rich AMPs) and interference with vital intracellular processes [47-50]. AMPs may elicit an anti-infective host immune response and possess the ability to neutralize endotoxins, suggesting potential efficacy in septic shock [42,51-54]. Importantly, their anti-biofilm properties may confer efficacy against infections associated with wounds, medical implants, and chronic respiratory illnesses. In contrast to standard antibiotics, AMPs have a number of properties that confer the ability to overcome common resistance mechanisms of MDR pathogens. (1) AMPs generally do not require metabolic processes for antimicrobial activity [55,56]; therefore, they are effective against both quiescent and actively growing bacteria. (2) They display rapid (seconds or minutes) killing kinetics, which would allow limited time for extensive growth and for mutations to occur [57]. (3) In sharp contrast to conventional antibiotics, AMPs demonstrate a low propensity to invoke selection of bacterial resistance [44,57-60]. Despite these unique properties, the clinical developments of AMPs have been largely unsuccessfully.

## Limitations of Natural AMPs

Several limitations of natural AMPs have delayed their successful development for clinical use. Natural AMPs display (1) contextual activity with potential inhibition in the presence of acidic pH, saline, divalent cations, and serum or plasma [57,60]; (2) insufficient evidence for systemic efficacy in animal models, which is particularly important in the context of sepsis as a complication of MDR-associated wound infections; (3) potential for susceptibility to protease digestion, which may limit their applications; (4) unclear Pharmacokinetic (PK) properties; (5) potential immunogenicity; (6) potential host toxicity or safety concerns; (7) potential resistance to AMPs, although far less common than resistance to current antibiotics (*e.g.*, by lipid modification toward a reduced density of negative charges) [61,62]. These limitations can be overcome by structural design optimization, as shown by studies of engineered peptides [20,37,57,60].

## Structural Optimization of AMPs

Inherently, AMPs are more preventive than therapeutic and are not dedicated antibiotics, whereas antibiotics typically cure diseases. AMPs have evolved to perform multiple functions, which may explain the diversity in amino acid compositions. However, as we (and others) have shown, a diverse amino acid composition is not essential for antimicrobial activity and may even interfere with structural optimization for antibiotic function. Therefore, it is important to approach AMPs differently from the way we approach discoveries of standard antibiotics to optimize their structures based on how they work. In that regard, several studies have already demonstrated that AMPs can be best optimized by reducing the diversity in amino acid composition and focusing mainly on the incorporation of cationic and hydrophobic amino acids, which are more specifically responsible for the amphipathic structure and antimicrobial properties. Such an approach has resulted in optimized AMPs that retain activities in environments of high ionic strength, low pH, and may even work systemically. This is the case of the engineered AMP WLBU2, which demonstrates efficacy in a *P. aeruginosa* septicemia model when systemically administered. WLBU2 is also very effective against *P. aeruginosa* when directly delivered to the airway in a murine pneumonia model [63]. WLBU2 is composed of only three amino acids (Arg, Val, and Trp) and designed to fold into an idealized amphipathic helix. In addition, AMPs can be modified to enhance PK properties by D-amino acid substitution [64,65], AMP mimic (e.g., peptoids) [66], cyclization [67], and end-terminus modification [46,68,69].

## Anti-Biofilm Properties of AMPS

For SSI, anti-biofilm properties are crucial. When bacteria grow in rich nutrient broth in the laboratory, they grow rapidly (exponentially) in a planktonic mode until the nutrients in the growth medium are largely consumed. However, some bacteria do not grow under such conditions or may adopt an alternate mode of growth called biofilm depending on the growth condition. Bacteria have the capacity to attach to surfaces and secrete an extracellular matrix as a shield against stringent environmental conditions. Similarly, bacteria tend to colonize surfaces of human tissues and adopt a biofilm mode of growth, which is often much less susceptible to antibiotics than bacteria growing in planktonic form. The property to form biofilm enhances the ability of bacteria to “escape” standard treatment, and trauma or post-surgical patients are at high risk of developing these types of infections. Several AMPs display antibiofilm properties, and anti-biofilm prevention and disruption properties can be enhanced in engineered AMPs such as WLBU2 and others [5,20,70]. Appropriate SSI *in vivo* testing models remain to be developed for further advancement of AMPs for the treatment of SSI and other biofilm-related infections.

## Concluding Remarks

The field of AMPs has been developed for more than three decades, but its success at a clinical level is still negligible. While numerous studies have addressed the limitations of AMPs, they are either scattered or incremental. Based on characteristics of AMPs (especially designed or modified AMPs), they hold a great promise to overcome MDR bacteria-associated SSI. To establish AMPs as a reliable source of effective therapeutics, systematic studies are needed to serve as a reference for multiple groups of investigators dedicated to the continuous development of AMPs.
